# Ophiostomatalean Fungi (Ascomycota, Ophiostomatales) Associated with Three Beetles from *Pinus sylvestris* var. *mongolica* in Heilongjiang, China

**DOI:** 10.3390/jof11010027

**Published:** 2025-01-02

**Authors:** Zheng Wang, Caixia Liu, Yingjie Tie, Xiuyue Song, Huimin Wang, Quan Lu

**Affiliations:** 1 College of Plant Protection, Shandong Agricultural University, Tai’an 271018, China; zhengwang@sdau.edu.cn (Z.W.); 18297112106@163.com (Y.T.); 13563501883@163.com (X.S.); 2 Key Laboratory of Forest Protection of National Forestry and Grassland Administration, Ecology and Nature Conservation Institute, Chinese Academy of Forestry, Beijing 100091, China; luicx123@126.com (C.L.); wanghuimin@caf.ac.cn (H.W.)

**Keywords:** forest decline, *Graphilbum*, *Ophiostoma*, pine, symbiosis

## Abstract

Globally, forest decline and tree mortality are rising due to climate change. As one of the important afforestation trees in northeast China, *Pinus sylvestris* var. *mongolica* is suffering from forest decline and the accompanying pests. Certain fungi from the ophiostomatalean contribute to forest pest outbreaks and can be pathogenic to pine trees. However, only a limited number of ophiostomatalean fungi associated with beetles infesting *Pinus sylvestris* var. *mongolica* have been identified. In this study, 293 ophiostomatalean fungi were isolated from *Acanthocinus griseus*, *Ips chinensis*, and *Pissodes nitidus* infesting *Pinus sylvestris* var. *mongolica* in Heilongjiang Province, including *Graphilbum griseum* sp. nov., *Gra. nitidum* sp. nov., *Graphilbum* sp., and *Ophiostoma ips*. *Ophiostoma ips* was the dominant species, followed by *Graphilbum* sp., *Graphilbum griseum*, and *Gra. nitidum*, which accounted for 73.38, 17.41, 7.17, and 2.05% of the isolated ophiostomatalean fungi, respectively. Fungi associated with different beetles are diverse, even within the same host. This study deepens our understanding of the pest-associated fungi of *P. sylvestris* var. *mongolica* and provides a basis for exploring the causes of forest decline.

## 1. Introduction

Climate change has led to increased forest decline and tree mortality worldwide [1]. Mongolian Scots pine (*Pinus sylvestris* var. *mongolica*) is a variety of Scots pine (*Pinus sylvestris*) mainly distributed in northeast China [2]. As one of the main afforestation species in the Three North Protective Forest Program (Green Great Wall), planting areas of *P. sylvestris* var. *mongolica* have exceeded 3.0×10^5^ ha [3]. Recently, a decline in the population of *P. sylvestris* var. *mongolica* plantations has been observed, with outbreaks of insect pests and diseases accompanied by climate change being considered an important factor [4].

Boring insects, such as bark beetles, weevils, and longhorned beetles, are major pests affecting pine trees and are prevalent in China [5]. Microorganisms associated with these insects, especially ophiostomatalean fungi, are beneficial for the development of beetle vectors and play a key role in tree mortality. Ophiostomatales belong to Sordariomycetidae of Ascomycota, containing more than 400 species in 20 genera, and are common beetle associates [6,7,8,9,10,11]. For example, *Leptographium clavigerum,* associated with *Dendroctonus ponderosae*, assists beetles by detoxifying the host tree’s chemical defenses and causing canker staining in *Pinus contorta* [12]. *Leptographium procerum* is considered an important factor in the successful invasion of its vector *Dendroctonus valens* in China, acting as a lethal pathogen responsible for pine root diseases [13]. Thus, understanding beetle–fungal associations is crucial for studying forest pest outbreaks.

In northeastern China, a total of 62 ophiostomatalean fungi-associated bark beetles have been reported [10]. While 39 species were associated with beetles infesting pine trees, only three were isolated from *P. sylvestris* var. *mongolica*. Among these, *Ophiostoma minus* is widely distributed in the northern hemisphere and is associated with a variety of bark beetles, such as *Dendroctonus* spp., *Tomicus* spp., and *Ips* spp., whereas *Ceratocystiopsis subelongati* and *Masuyamyces lotiformis* are endemic and only associated with *Ips subelongatus* from *P. sylvestris* var. *mongolica* in Inner Mongolia [14,15]. The limited research on ophiostomatalean fungi associated with boring beetles infesting *P. sylvestris* var. *mongolica* has constrained the understanding of forest decline in this afforestation species.

In the present study, *P. sylvestris* var. *mongolica* trees infested by beetles were surveyed in Heilongjiang Province through 2023. Fungal isolates from three beetle species were identified based on their morphological features and multigene phylogenetic analyses. This study provides new insights into the diversity of ophiostomatalean fungi associated with *P. sylvestris* var. *mongolica* and advances understanding of the decline in plantations of this tree.

## 2. Materials and Methods

### 2.1. Sample Collection and Fungal Isolation

In June 2023, *Pinus sylvestris* var. *mongolica* with a withered crown and scattered sawdust and beetle feces on the trunk were collected from a pine plantation in Huanan County, Heilongjiang Province. The trunk close to the ground was cut into one-meter-long logs and transported to the laboratory, where they were stored at room temperature. The discoloration around the beetle tunnels was observed after peeling off some bark. Over the next two months, adults of three beetle species, *Acanthocinus griseus*, *Ips chinensis*, and *Pissodes nitidus*, emerged from the logs. Ten adults of each species were used for fungal isolation. We did not identify the sex of the beetles nor find their mycangia. Depending on the size of the adult beetles, they were separated into 15 to 40 tissue pieces, which were placed on 2% water agar (20 g agar and 1 L distilled water) and incubated in the dark at 25 °C until mycelium grew from the sample. A single hyphal tip was transferred to 2% malt extract agar (MEA, 20 g malt extract, 20 g agar, and 1 L distilled water) medium in the dark at 25 °C to obtain a pure culture.

### 2.2. Morphological Analyses

Conidia and conidiogenous structures were observed and recorded using an Olympus BX43 microscope (Olympus Corporation, Tokyo, Japan) equipped with a BioHD-A20c color digital camera (FluoCa Scientific, Shanghai, China). Thirty conidia and conidiogenous structures were randomly selected, and their lengths and widths were measured and recorded. Five millimeter agar plugs were transferred onto the center of a 90-mm-diameter Petri plate containing 2% MEA in the dark at 25 °C from the actively growing colonies to observe and record cultural characteristics. To assess the optimal growth temperature, the cultures were incubated at temperatures ranging from 5 °C to 40 °C at 5 °C intervals in darkness.

### 2.3. DNA Extraction, PCR Amplification, and Sequencing

Actively growing mycelia were collected for DNA extraction using a Fungal Genomic DNA Extraction Kit (Solarbio Co., Ltd., Beijing, China), according to the manufacturer’s instructions. Three gene fragments—the internal transcribed spacer regions 1 and 2 of the nuclear ribosomal DNA operon, including the 5.8S region (ITS), β-tubulin gene region (*tub2*), and transcription elongation factor 1-α gene region (*tef1-α*)—were amplified using the primer pairs with different conditions, as listed in Table 1. The 2 × Taq PCR Master Mix (Tiangen Biotech Co., Ltd., Beijing, China) was used for PCR amplification following the manufacturer’s instructions. Sequencing of the PCR products was conducted by Rui Biotech Co., Ltd. (Beijing, China).

### 2.4. Phylogenetic Analysis

Newly obtained sequences were subjected to a standard nucleotide BLAST search through the National Center for Biotechnology Information (NCBI) to determine species affinities. Reference sequences were selected based on previous publications and were downloaded from GenBank. Alignments were performed using the MAFFT v.7 online web server [20] with default settings and then edited and improved manually using MEGA 7.0 [21]. Maximum likelihood (ML) phylogenetic analyses were performed using RAxML-HPC v.8.2.3 [22] in the GTRGAMMA model with 1000 replicates. MrBayes v. 3.1.2 [23] was used for Bayesian inference (BI) under the best substitution models, which were determined using jModelTest v.2.1.7 [24]. Four Markov chain Monte Carlo chains were run simultaneously from a random starting tree for 5,000,000 generations.

## 3. Results

### 3.1. Sampling Collection and Fungal Isolation

In the present study, ten adult beetles of *Acanthocinus griseus*, *Ips chinensis*, and *Pissodes nitidus* from *Pinus sylvestris* var. *mongolica* were used for fungal isolation. In total, 157, 59, and 77 ophiostomatalean strains were isolated from these beetles, respectively. Of these, 13 representative strains were used for phylogenetic analyses (Table 2).

### 3.2. Phylogenetic Analysis

**Figure 1:**
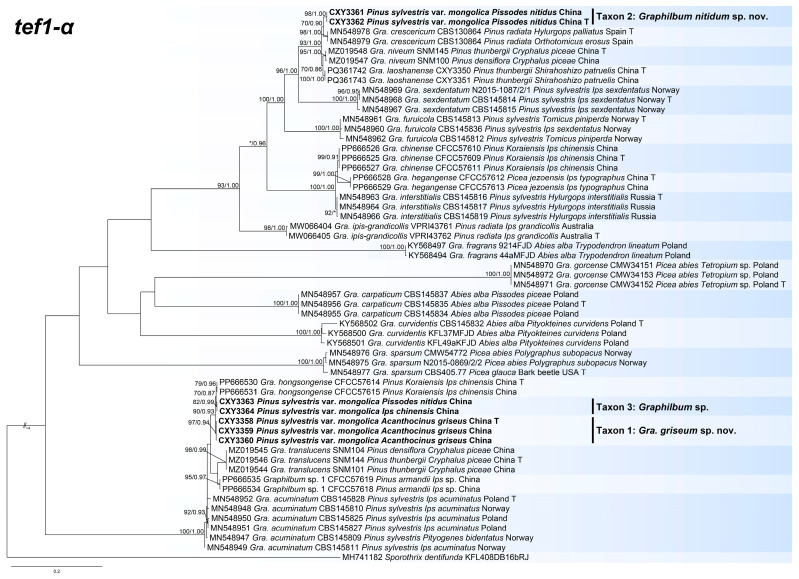
Maximum likelihood (ML) tree based on *tef1-α* sequence data of *Graphilbum* spp. ML bootstrap values above 70% and Bayesian posterior probability values above 0.85 are indicated at the nodes. Sequences generated from this study are shown in bold. T = ex-type.

We performed phylogenetic inferences for *Graphilbum* using the ITS, *tub2*, and *tef1-α* datasets. The alignments of the three datasets contained 570, 574, and 875 characters, respectively (including gaps). The best evolutionary models were GTR+G (ITS dataset) and HKY+I+G (*tub2* and *tef1-α* datasets). In the ITS phylogenetic tree (Figure S1), our seven isolates represented three taxa (taxa 1–3). Among these, taxon 1, taxon 3, *Gra. acuminatum*, and *Gra. hongsongense* formed a monophyletic clade with *Gra. xianjuensis*, *Graphilbum* sp. 1, *Gra. anningense*, *Gra. translucens*, and *Gra. puerense*. Taxon 2 formed a well-supported terminal clade. In the *tub2* tree (Figure S2), taxon 2 formed a branch between the *Gra. niveum* and *Gra. laoshanense* clades. The *tef1-α* sequence provided the highest-resolution DNA barcode for dividing species boundaries in *Graphilbum*. In the *tef1-α* phylogenetic tree (Figure 1), our seven isolates formed three well-supported terminal clades, representing three undescribed taxa. Taxon 1 was a phylogenetic sister to *Gra. hongsongense* and *Graphilbum* sp.; it formed a subclade with *Gra. translucens* and *Graphilbum* sp. 1. Taxon 2 was a phylogenetic sister to *Gra. crescericum* and formed a subclade with *Gra. niveum* and *Gra. laoshanense*.

The ITS and *tub2* datasets were used to perform a phylogenetic analysis of the *Ophiostoma ips* complex. The alignments of these datasets contained 624 and 271 characters (including gaps). The best evolutionary models of the ITS and *tub2* datasets were GTR+G and HKY+I, respectively. Our six isolates clustered in a clade with known isolates of *O. ips* based on the ITS and *tub2* trees (Figures S3 and S4).

### 3.3. Taxonomy

**Figure 2:**
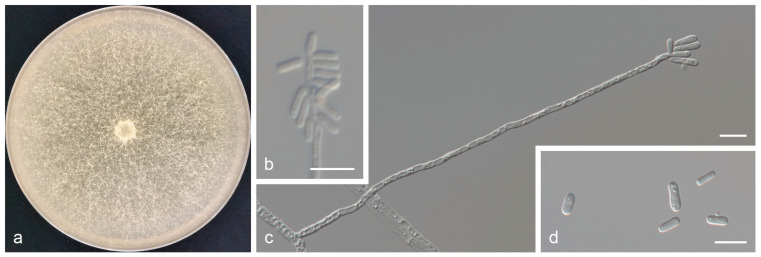
*Graphilbum griseum* sp. nov. (**a**) Eight-day-old culture on 2% MEA; (**b**–**d**) *Hyalorhinocladiella*-like asexual morph: conidiogenous cells and conidia. Scale bars of (**b**–**d**) = 10 μm.

*Graphilbum griseum* Z. Wang & Q. Lu, sp. nov. (Taxon 1 Figure 2).

Mycobank: 856720

Etymology: The epithet *griseum* (Latin) refers to its vector, *Acanthocinus griseus*.

Holotype: CXY3358

*Description*: Sexual morph: not observed. Asexual morph: *hyalorhinocladiella*-like. *Conidiophores* are simple, arising directly from the mycelium; *conidiogenous cells* are subulate, hyaline, smooth or rough, (13.9–)23.4–53.8(–69.8) × (2.0–)2.2–3.1(–3.6) μm. *Conidia* are hyaline, smooth, aseptate, cylindrical to obovate, (5.8–)5.9–8.7(–11.5) × (2.4–)2.5–3.2(–3.7) μm.

*Culture characteristics*: Colony diameters reached 78.7 mm in seven days on 2% MEA at 25 °C. Colonies were initially hyaline or light white. Mycelia later became grayish-white and superficial, with abundant aerial mycelia. Radial thinning of the colony margins. Colonies grow fastest at 30 °C and do not grow at 5 °C or 40 °C.

*Associated insects: Acanthocinus griseus*.

*Hosts*: *Pinus sylvestris* var. *mongolica*.

*Material examined*: CHINA, Heilongjiang Province, Jiamusi City, Huanan County, from *Acanthocinus griseus* infesting *Pinus sylvestris* var. *mongolica*, June 2023, Z. Wang and Q. Lu, holotype: CXY3358, ex-type culture CFCC71126, ibid. CFCC71130, CFCC71124.

*Notes*: *Graphilbum griseum* was closely related to *Gra. acuminatum* and *Gra. hongsongense* in phylogenetic inferences (Figure 1 and Figures S1 and S2) [10,25]. Although these three species had an identical ITS sequence (Figure S1), they were distinct species based on phylogenetic analysis of *tef1-α* sequences (Figure 1). *Graphilbum griseum* can be distinguished from *Gra. acuminatum* and *Gra. hongsongense* by a *hyalorhinocladiella*-like asexual morph, which is *pesotum*-like in *Gra. acuminatum* and *Gra. hongsongense*. The optimal growth temperatures for the three species were 30 °C (*Gra. griseum*) and 25 °C (*Gra. acuminatum* and *Gra. hongsongense*). At 25 °C on 2% MEA, *Gra. griseum* grew slower than *Gra. hongsongense* (78.7 mm in 7 days vs. 76.3 mm in 4 days) and faster than *Gra. acuminatum* (11.2 mm/d vs. 5.8 mm/d). Furthermore, *Gra. griseum* was associated with *Acanthocinus griseus* from *Pinus sylvestris* var. *mongolica* in Jiamusi, China, whereas *Gra. hongsongense* was associated with *Ips chinensis* from *Pinus koraiensis* in Hegang, China. *Graphilbum acuminatum* used *Pinus sylvestris* and *Picea abies* as hosts and was associated with *Ips acuminantus*, *Orthotomicus laricis*, *Pityogenes bidentatus*, and *Pityogenes quadridens* in Europe.

**Figure 3:**
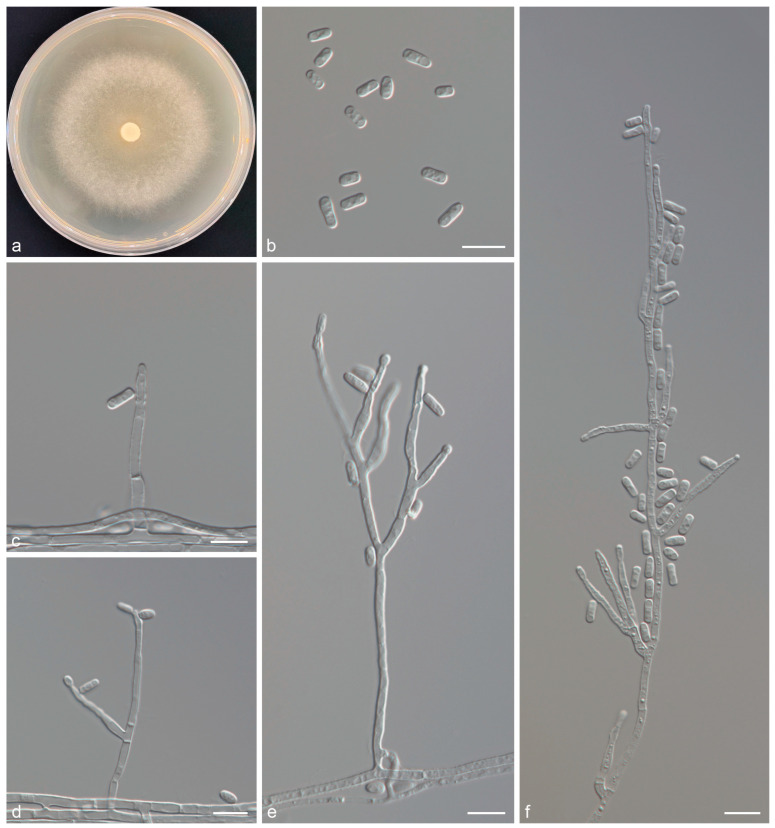
*Graphilbum nitidum* sp. nov. (**a**) Eight-day-old culture on 2% MEA; (**b**–**f**) *Hyalorhinocladiella*-like asexual morph: conidiogenous cells and conidia. Scale bars of (**b**–**f**) = 10 μm.

*Graphilbum nitidum* Z. Wang & Q. Lu, sp. nov. (Taxon 2 Figure 3).

Mycobank: 856721

Etymology: The epithet *nitidum* (Latin) refers to its vector, *Pissodes nitidus*.

Holotype: CXY3362

*Description*: Sexual morph: not observed. Asexual morph: *hyalorhinocladiella*-like. *Conidiophores* are simple or sparingly branched, arising directly from the mycelium; *conidiogenous cells* are sympodial, subulate, hyaline, 1–3 per branch, smooth or rough, (11.7–)19.1–35.5(–47.5) × (1.9–)2.1–2.8(–3.4) μm. *Conidia* are hyaline, smooth, aseptate, cylindrical to obovate, (4.7–)5.3–6.6(–7.6) × (2.5–)2.7–3.2(–3.5) μm.

*Culture characteristics*: Colony diameters reached 40.2 mm in eight days on 2% MEA at 25 °C. Colonies were initially hyaline or light white. Mycelia later became grayish white and superficial, with aerial mycelia. Radial thinning of the colony margins. Colonies grow fastest at 30 °C and do not grow at 5 °C and 40 °C.

*Associated insects*: *Pissodes nitidus*.

*Hosts*: *Pinus sylvestris* var. *mongolica*.

*Material examined*: CHINA, Heilongjiang Province, Jiamusi City, Huanan County, from *Pissodes nitidus* infesting *Pinus sylvestris* var. *mongolica*, June 2023, Z. Wang and Q. Lu, holotype: CXY3362, ex-type culture CFCC71143, ibid. CFCC71144.

*Notes*: *Graphilbum nitidum* was phylogenetically close to *Gra. crescericum* (Figure 1 and Figures S1 and S2), both of which were observed in a *hyalorhinocladiella*-like asexual morph [26]. There were differences in conidial shape and size (*Gra. nitidum*: cylindrical to obovate, 5.3–6.6 μm long; *Gra. crescericum*: globose-subglobose, 4.5–5.7 μm long). The optimal growth temperatures for the two species were 30 °C (*Gra. nitidum*) and 25 °C (*Gra. crescericum*). At 25 °C on 2% MEA, *Gra. nitidum* grew slower than *Gra. crescericum* (8 days: 40.2 mm vs. 60.4 mm). In addition, *Gra. nitidum* was associated with *Pissodes nitidus* from *Pinus sylvestris* var. *mongolica* in China, whereas *Gra. crescericum* was isolated from *Hylurgops palliatus*, *Hylastes ater*, and *Orthotomicus erosus*, infesting *Pinus radiata* in Europe [25,26].

## 4. Discussion

The results of this study showed that four ophiostomatalean fungi were associated with three beetles infesting *Pinus sylvestris* var. *mongolica*, including *Graphilbum griseum* sp. nov., *Gra. nitidum* sp. nov., *Graphilbum* sp., and *Ophiostoma ips* (Table 2 and Table 3). The dominant species was *O. ips*, with an isolation rate of 73.38%, a fungus commonly found in pine forests worldwide [27]. In China, *O. ips* is widely distributed, associated with various pine-infesting bark beetles, and has also been isolated from pine trees infested by *Bursaphelenchus xylophilus* [7,28,29,30,31]. In this study, *O. ips* was obtained from all three beetles, viz. *Acanthocinus griseus*, *Ips chinensis*, and *Pissodes nitidus* (Table 3). However, there is no evidence of specificity between *O. ips* and particular beetle vectors [27].

The genus *Graphilbum,* belonging to the family Ophiostomataceae, is commonly associated with conifer-infesting bark beetles [6,25]. This study identified two new species of *Graphilbum*, increasing the total number of species described in this genus to 32 [6,7,9,10,32]. *Graphilbum griseum* and *Gra. nitidum* were specific associated with *A. griseus* and *P. nitidus*, respectively, whereas *Graphilbum* sp. was associated with *I. chinensis* and *P. nitidus* (Table 3). The three beetles occupy different ecological niches within the trunk, with *A. griseus* living in the xylem, and *I. chinensis* and *P. nitidus* in the bark. This distinction may explain the differences in the fungal species associated with these beetles. Furthermore, species-specific associations between *Ips* bark beetles and ophiostomatoid fungi have been observed [10], and similar specific associations may exist between beetles of different families and these fungi. However, a larger sample size is necessary to confirm this hypothesis.

There may also be community succession among fungi associated with beetles. Zheng et al. found that the fungal communities associated with the 2nd–3rd- and 4th–5th-instar larvae of *Monochamus alternates* differed [32]. Fungal associates of 4th-5th-instar larvae were fewer than those of 2nd–3rd-instar larvae, with 14 and 6 species, respectively, and only two shared species. Sixteen ophiostomatalean fungi have been reported to be associated with *Ips chinensis*, including eight from northeast China [10,29]. However, the two ophiostomatalean fungal associates of *I. chinensis* in the present study did not overlap with these eight. Compared with the findings of Wang et al. [10], this study only used emerging adults for fungal isolation, which may explain why only two associates of *I. chinensis* were identified. Thus, fungal isolation across different beetle lifestyles can better reflect the associated fungal communities.

Beetles are known to carry various tree disease pathogens. For example, the diplodia tip blight pathogen of *P. sylvestris* var. *mongolica*, *Diplodia sapinea*, is the dominant fungus associated with *Pityophthorus morosovi*. Additionally, the associate of *Ips subelongatus* infesting *P. sylvestris* var. *mongolica*, *Ceratocystiopsis subelongati*, has been found to be pathogenic to pine trees [14]. Interestingly, *Ophiostoma minus*, which causes canker staining in pines, has been detected in the endophytic fungal communities of *P. sylvestris* var. *mongolica* infected with diplodia tip blight [33]. Therefore, pathogenicity tests of the fungi identified in this study are essential to assess their role in the decline of *P. sylvestris* var. *mongolica*.

## 5. Conclusions

In summary, four ophiostomatalean fungal associates from three beetles (*A. griseus*, *I. chinensis*, and *P. nitidus*) infesting *P. sylvestris* var. *mongolica* were identified in this study, including *Graphilbum griseum*, *Gra. nitidum*, *Graphilbum* sp., and *O. ips*. The results of this study revealed that the fungi associated with different beetles are diverse, even when they share the same host. Moreover, the correlation between ophiostomatalean fungi and forest decline needs to be further studied by means of, for example, pathogenicity tests.

## Figures and Tables

**Table 1:** Primers and PCR conditions used in this study.

Gene Fragment	Primers	PCR Conditions	References
ITS	ITS1-F/ITS4	94 °C for 3 min, 35 cycles of 94 °C for 1 min, 55 °C for 45 s, and 72 °C for 1 min, 72 °C for 8 min	[16,17]
*tub2*	Bt2a/Bt2b	95 °C for 2 min, 35 cycles of 95 °C for 30 s, 56 °C for 30 s, and 72 °C for 1 min, 72 °C for 8 min	[18]
*tef1-α*	EF1F/EF2R	95 °C for 2 min, 35 cycles of 95 °C for 30 s, 56 °C for 30 s, and 72 °C for 1 min, 72 °C for 8 min	[19]

**Table 2:** Representative strains in this study.

Species	Taxon	Isolate No. ^1^	Other No. ^2^	Insect Vector ^3^	GenBank Accession No.		
					ITS	*tub2*	*tef1-α*
*Gra. griseum* sp. nov.	1	CFCC71126	CXY3358	*A. griseus*	PQ623400	-	PQ619704
		CFCC71130	CXY3359	*A. griseus*	PQ623401	-	PQ619705
		CFCC71124	CXY3360	*A. griseus*	PQ623402	-	PQ619706
*Gra. nitidum* sp. nov.	2	CFCC71144	CXY3361	*P. nitidus*	PQ623403	-	PQ619707
		CFCC71143	CXY3362	*P. nitidus*	PQ623404	PQ619697	PQ619708
*Graphilbum* sp.	3	CFCC71127	CXY3363	*P. nitidus*	PQ623405	-	PQ619709
		CFCC71139	CXY3364	*I. chinensis*	PQ623406	-	PQ619710
*Ophiostoma ips*	4	CFCC71129	CXY3365	*A. griseus*	PQ623407	PQ619698	-
		CFCC71135	CXY3366	*A. griseus*	PQ623408	PQ619699	-
		CFCC71128	CXY3367	*I. chinensis*	PQ623409	PQ619700	-
		CFCC71136	CXY3368	*I. chinensis*	PQ623410	PQ619701	-
		CFCC71137	CXY3369	*P. nitidus*	PQ623411	PQ619702	-
		CFCC71138	CXY3370	*P. nitidus*	PQ623412	PQ619703	-

^1^ CFCC: the China Forestry Culture Collection Center. ^2^ CXY: the culture collection of the Forest Pathology Laboratory at the Chinese Academy of Forestry. ^3^ *A*.: *Acanthocinus*; *I*.: *Ips*; *P.*: *Pissodes*.

**Table 3:** Isolates from *Pinus sylvestris* var. *mongolica* infested by three beetles in this study.

Taxon	Species	Numbers of Isolates ^1^			Total	Total Percentage
		*A. griseus*	*I. chinensis*	*P. nitidus*		
1	*Graphilbum griseum*	21			21	7.17%
2	*Gra. nitidum*			6	6	2.05%
3	*Graphilbum* sp.		25	26	51	17.41%
4	*Ophiostoma ips*	136	34	45	215	73.38%
Total		157	59	77	293	100.00%

^1^ A.: Acanthocinus; I.: Ips; P.: Pissodes.

## Data Availability

The data presented in this study are openly available in [GenBank].

## Supplementary Materials

The following supporting information can be downloaded at: https://www.mdpi.com/article/10.3390/jof11010027/s1, Figure S1: Maximum likelihood (ML) tree based on ITS sequence data of *Graphilbum* spp. ML bootstrap values above 70% and Bayesian posterior probability values above 0.85 are indicated at the nodes. Sequences generated from this study are shown in bold. T = ex-type; Figure S2: Maximum likelihood (ML) tree based on *tub2* sequence data of *Graphilbum* spp. ML bootstrap values above 70% and Bayesian posterior probability values above 0.85 are indicated at the nodes. Sequences generated from this study are shown in bold. T = ex-type; Figure S3: Maximum likelihood (ML) tree based on ITS sequence data of *Ophiostoma ips* complex. ML bootstrap values above 70% and Bayesian posterior probability values above 0.85 are indicated at the nodes. Sequences generated from this study are shown in bold. T = ex-type; Figure S4: Maximum likelihood (ML) tree based on *tub2* sequence data of *Ophiostoma ips* complex. ML bootstrap values above 70% and Bayesian posterior probability values above 0.85 are indicated at the nodes. Sequences generated from this study are shown in bold. T = ex-type.
