# Supplementary figures and images for: Ophiostomatalean Fungi (Ascomycota, Ophiostomatales) Associated with Three Beetles from Pinus sylvestris var. mongolica in Heilongjiang, China
Source: J Fungi (Basel). 2025 Jan 2;11(1):27. doi: 10.3390/jof11010027 (PMC11766559; doi:10.3390/jof11010027)

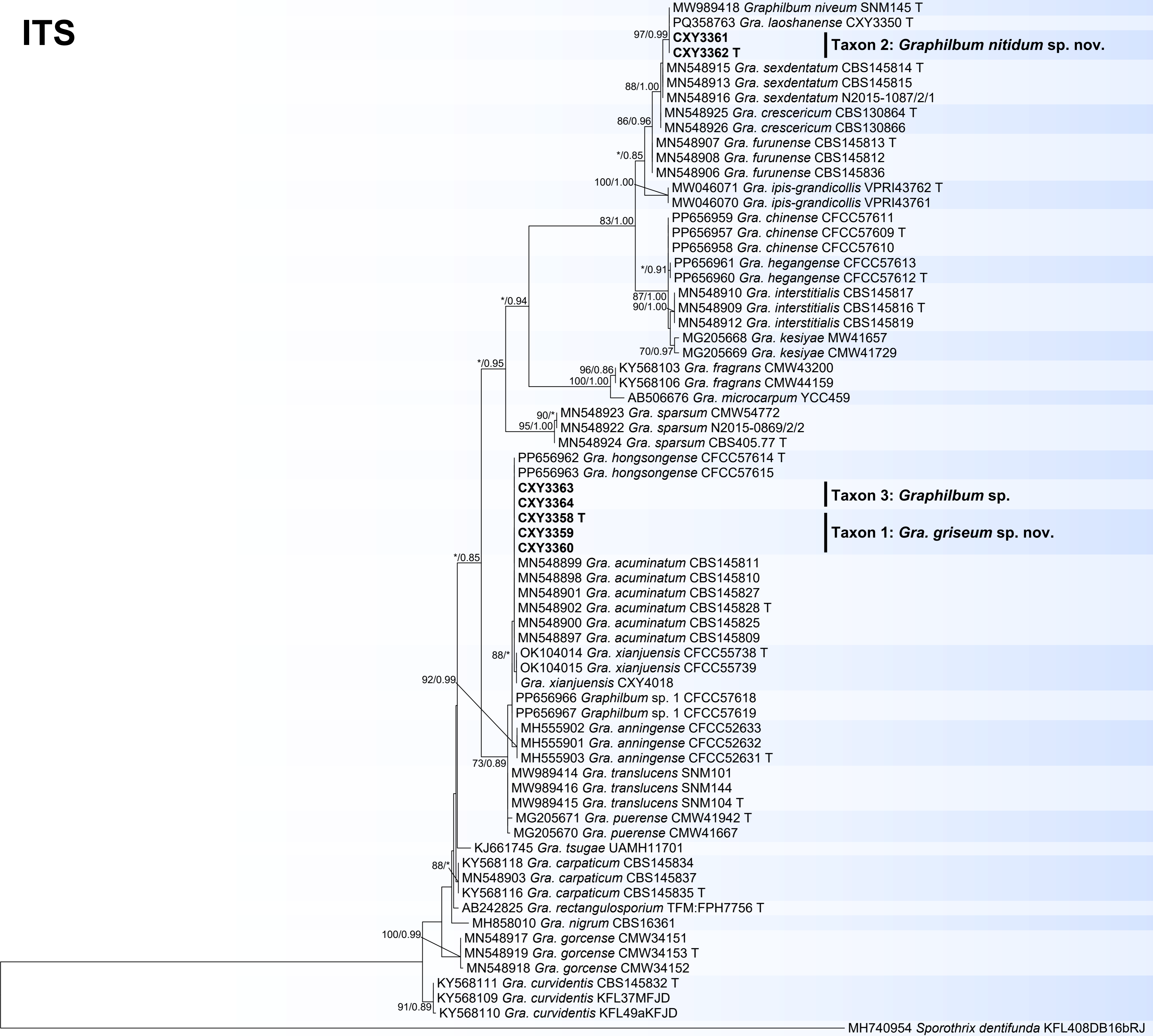

Supplement: Supplementary file 1 [file jof-11-00027-s001.zip › Figure S1.pdf]

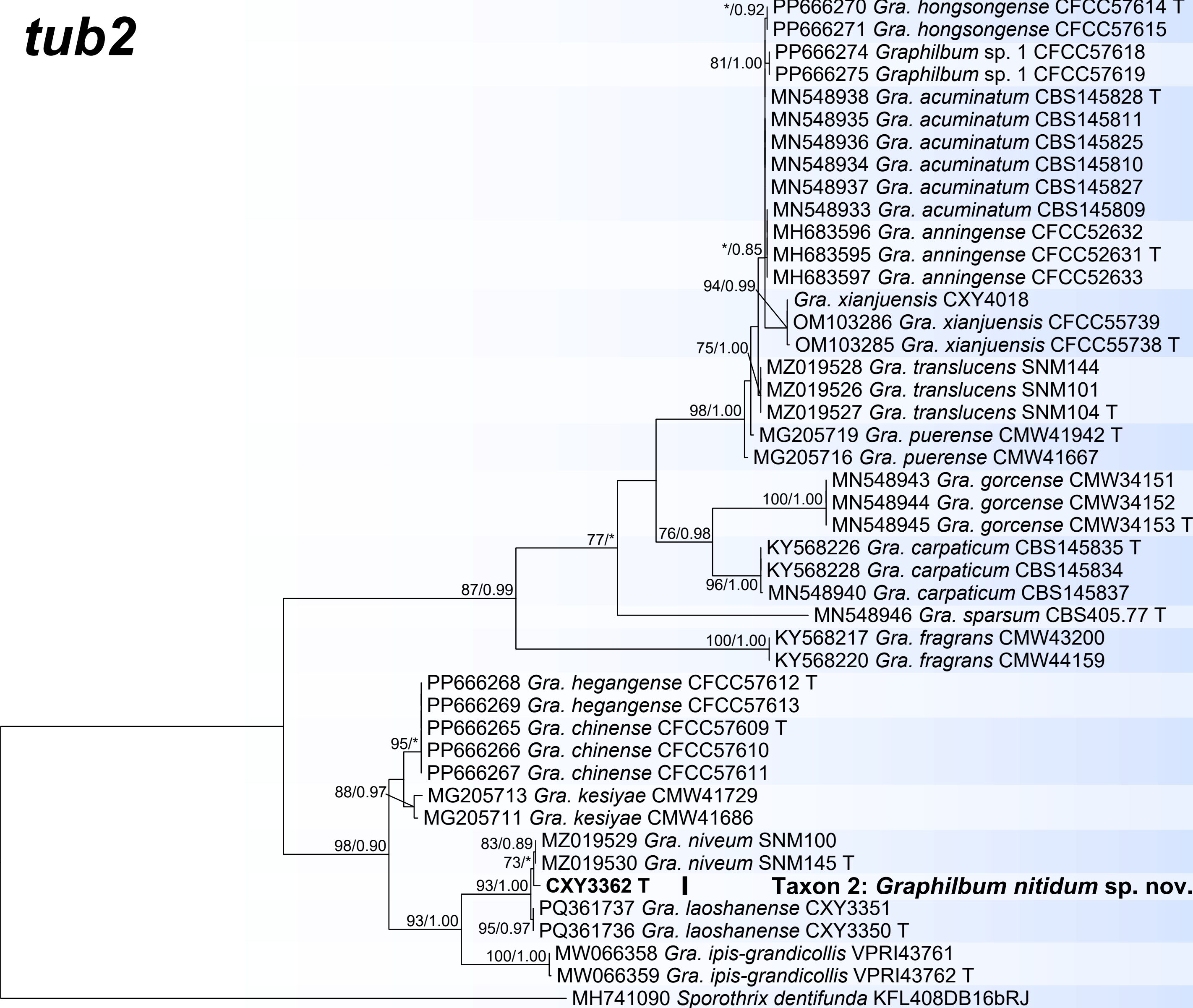

Supplement: Supplementary file 1 [file jof-11-00027-s001.zip › Figure S2.pdf]

ITS

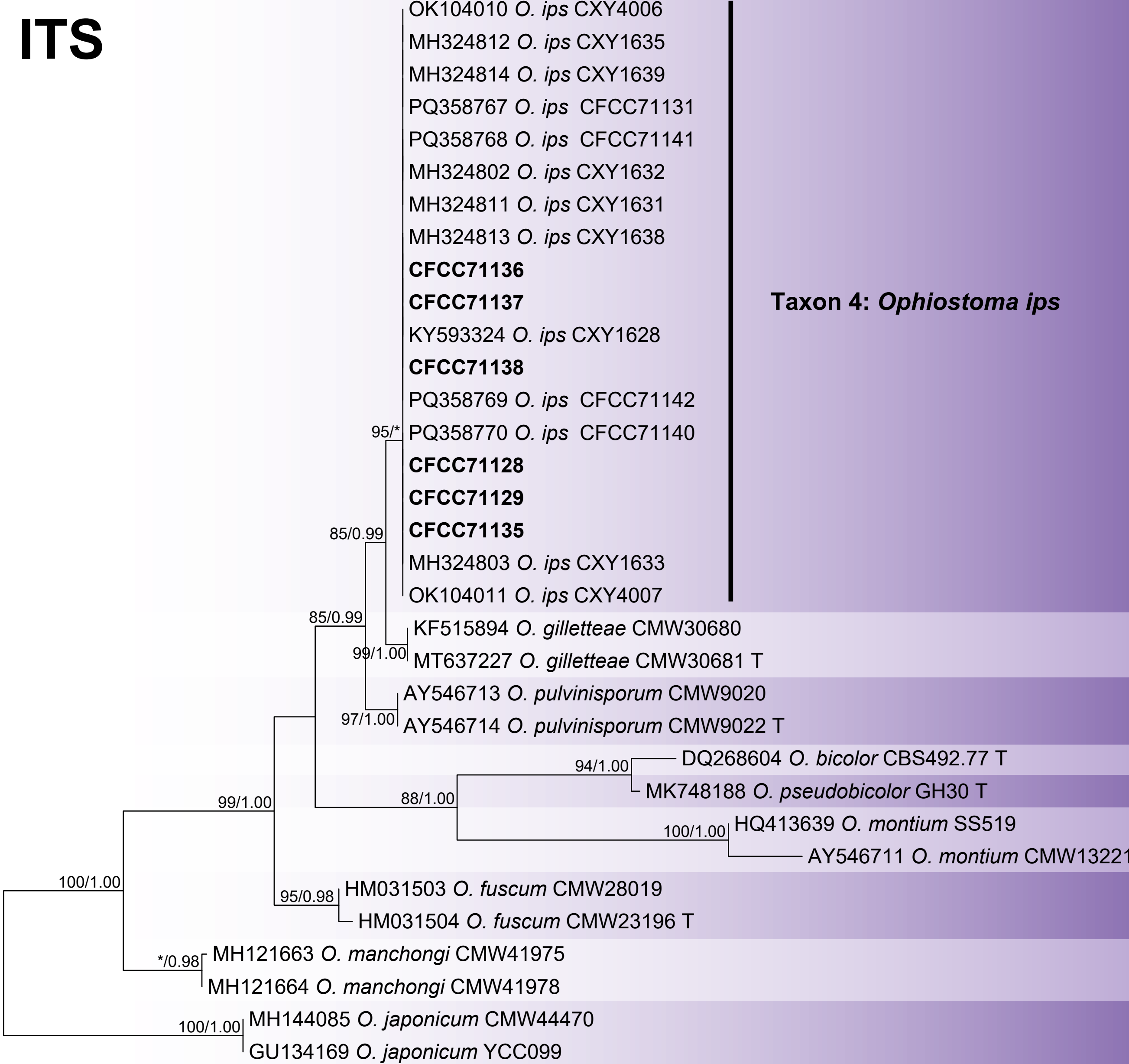

Taxon 4: *Ophiostoma ips*

Supplement: Supplementary file 1 [file jof-11-00027-s001.zip › Figure S3.pdf]

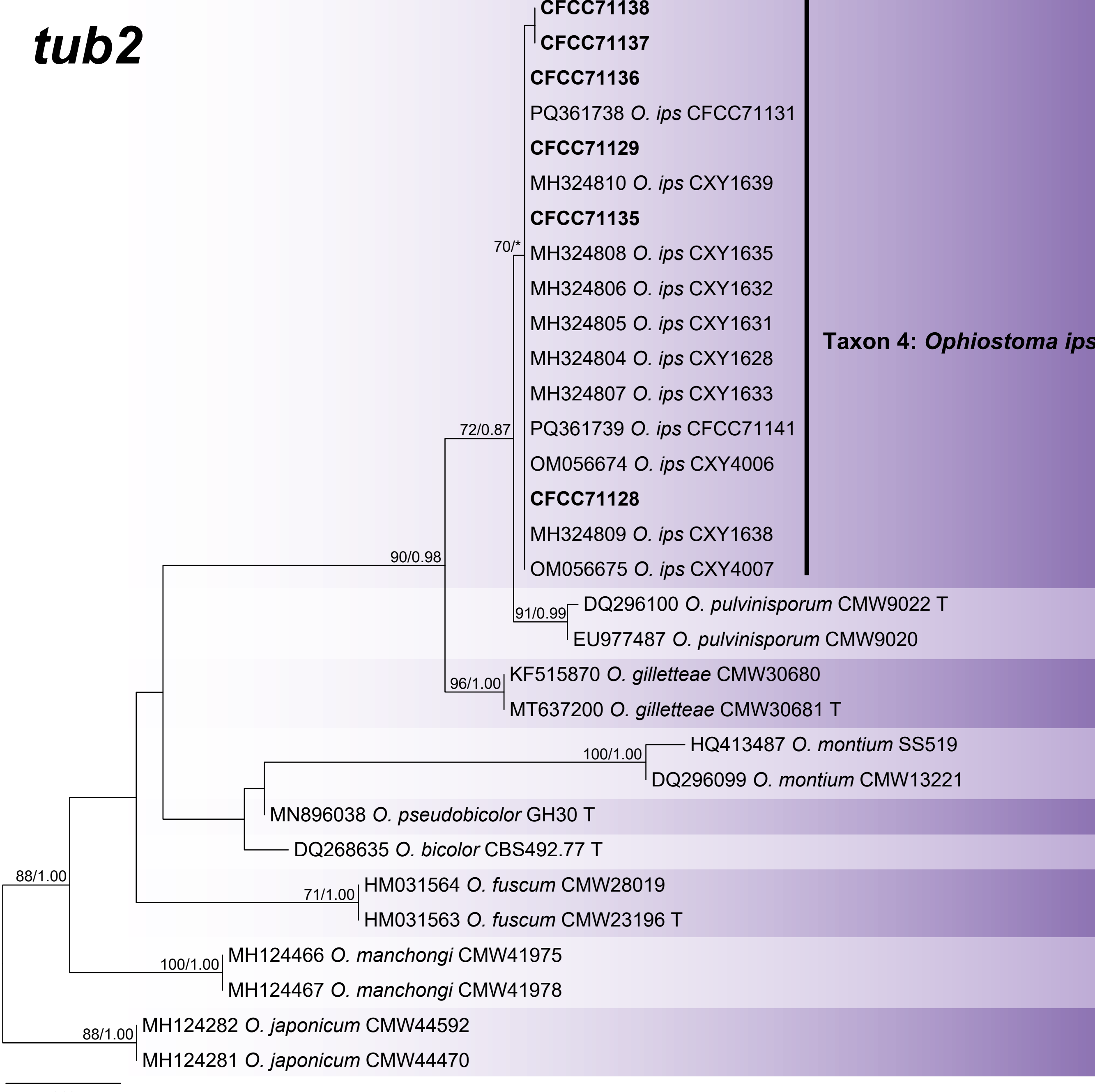

Supplement: Supplementary file 1 [file jof-11-00027-s001.zip › Figure S4.pdf]
